# Supplementary material for: Gel-Free Tools for Quick and Simple Screening of Anti-Topoisomerase 1 Compounds
Source: Pharmaceuticals (Basel). 2023 Apr 27;16(5):657. doi: 10.3390/ph16050657 (PMC10221459; doi:10.3390/ph16050657)
Supplement: Supplementary file 1 [file pharmaceuticals-16-00657-s001.zip › pharmaceuticals-2266619-supplementary.pdf]

pharmaceuticals

MDPI

Supplementary Materials

Gel-Free Tools for Quick and Simple Screening of Anti-Topoisomerase 1 Compounds

Josephine Geertsen Keller [1*], Kamilla Vandsø Petersen [2], Karol Mizielinski [1], Celine Thiesen [2], Lotte Bjergbæk [2], Rosa M Reguera [3], Yolanda Pérez-Pertejo [3], Rafael Balaña-Fouce [3], Angela Trejo [4], Carme Masdeu [4], Concepcion Alonso [4], Birgitta R. Knudsen [1] and Cinzia Tesauro [1,*#]

1 VPCIR Biosciences ApS, 8000 Aarhus C, Denmark
2 Department of Molecular Biology and Genetics, Aarhus University, 8000 Aarhus C, Denmark
3 Department of Biomedical Sciences, Faculty of Veterinary Medicine, University of León, 24071 León, Spain
4 Department of Organic Chemistry, Faculty of Pharmacy, University of Basque Country (UPV/EHU), 01006 Vitoria-Gasteiz, Spain
* Correspondence: ct@vpcir.com
* These authors contributed equally

Citation: Keller, J.G.; Petersen, K.V.; Mizielinski, K.; Thiesen, C.; Bjergbæk, L.; Reguera, R.M.; Pérez-Pertejo, Y.; Balaña-Fouce, R.; Trejo, A.; Masdeu, C.; et al. Gel-Free Tools for Quick and Simple Screening of Anti-Topoisomerase 1 Compounds. *Pharmaceuticals* **2023**, *16*, 657. https://doi.org/10.3390/ph16050657

Academic Editor: Andrej Perdih

Received: 21 February 2023
Revised: 21 April 2023
Accepted: 26 April 2023
Published: 27 April 2023

Copyright: © 2023 by the authors. Licensee MDPI, Basel, Switzerland. This article is an open access article distributed under the terms and conditions of the Creative Commons Attribution (CC BY) license (https://creativecommons.org/licenses/by/4.0/).

1. Supplementary S1

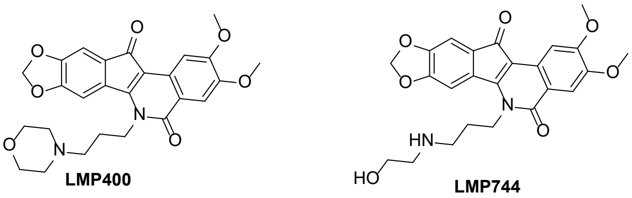

Figure S1. Chemical structure of Indotecan (LMP400, left) and LMP744 (right).

2. Supplementary S2

2.1. Protein purifications

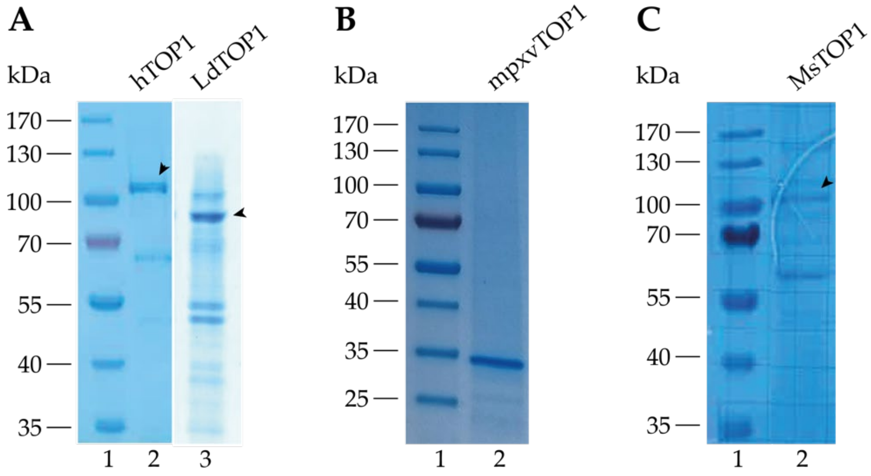

Figure S2. Coomassie stains of purified enzymes. **A**) Coomassie stain of purified fractions of hTOP1 (lane 2) and LdTOP1 (lane 3). Lane 1 is a size marker. The size of the bands is indicated to the left of the figure. Bands corresponding to hTOP1 and LdTOP1 are marked with an arrowhead. **B**) Coomassie stain of purified fraction of mpxvTOP1 (lane 2). Lane 1 is a size marker. The size of the bands is indicated to the left of the figure. **C**) Coomassie stain of purified fraction of MsTOP1 (lane 2). Lane

1 is a size marker. The size of the bands is indicated to the left of the figure. Band corresponding to MsTOP1 is marked with an arrowhead.

3. Supplementary S3

3.1. Dose dependent inhibition of hTOP1 and LdTOP1 activities detected using the REEAD assay

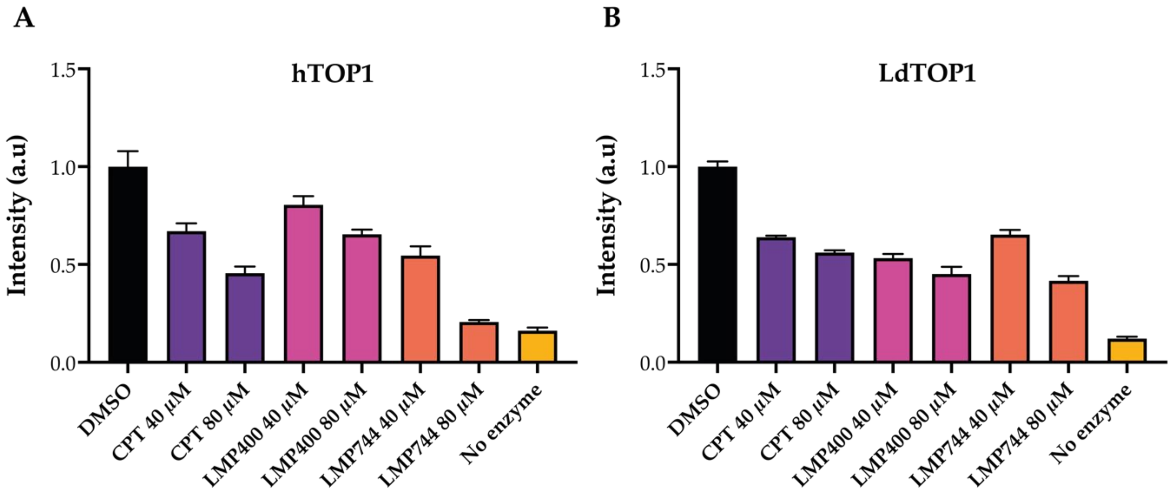

Figure S3. Dose dependent inhibition of hTOP1 and LdTOP1 activities. **A**) Graphical quantification of the results obtained when analyzing hTOP1 activity in the presence of 40-80 µM of the compounds CPT, LPM400, or LMP744 using REEAD with the ECL based readout as indicated on the figure. A negative control without enzyme was included. Plotted data are normalized to the intensity obtained when measuring hTOP1 activity in the presence of DMSO and represent average +/- standard error of the mean (SEM) from six independent experiments. a.u: arbitrary units. **B**) same as A, except that LdTOP1 was used instead of hTOP1. Plotted data represent average +/- SEM from three independent experiments. a.u: arbitrary units.

4. Supplementary S4

4.1. Synthesis of compounds targeting MsTOP1

As shown in **Figure S4A**, the synthesis of compounds has been carried out by the Povarov reaction between aromatic amine **1** (sulfadoxine) and different dienophiles such as styrene **3a** or acetylene **3b**, and indene **6** [1]. Directly, the three components, aromatic amine **1**, aldehydes **2** and the corresponding dienophiles **3** or **6** reacted in the presence of Lewis acid ($Yb(OTf)_3$). Thus, when styrene **3a** was used as a dienophile adducts **4** were obtained. Then, we proceeded to the *in-situ* aromatization of tetrahydroquinoline derivatives **4** by treatment with DDQ following published protocols [2] and corresponding derivatives **5** were isolated (**Figure S4B**). When acetylene **3b** was used, aromatic compounds **5** were directly isolated and when the dienophile used was indene **6**, adduct **7** was isolated.

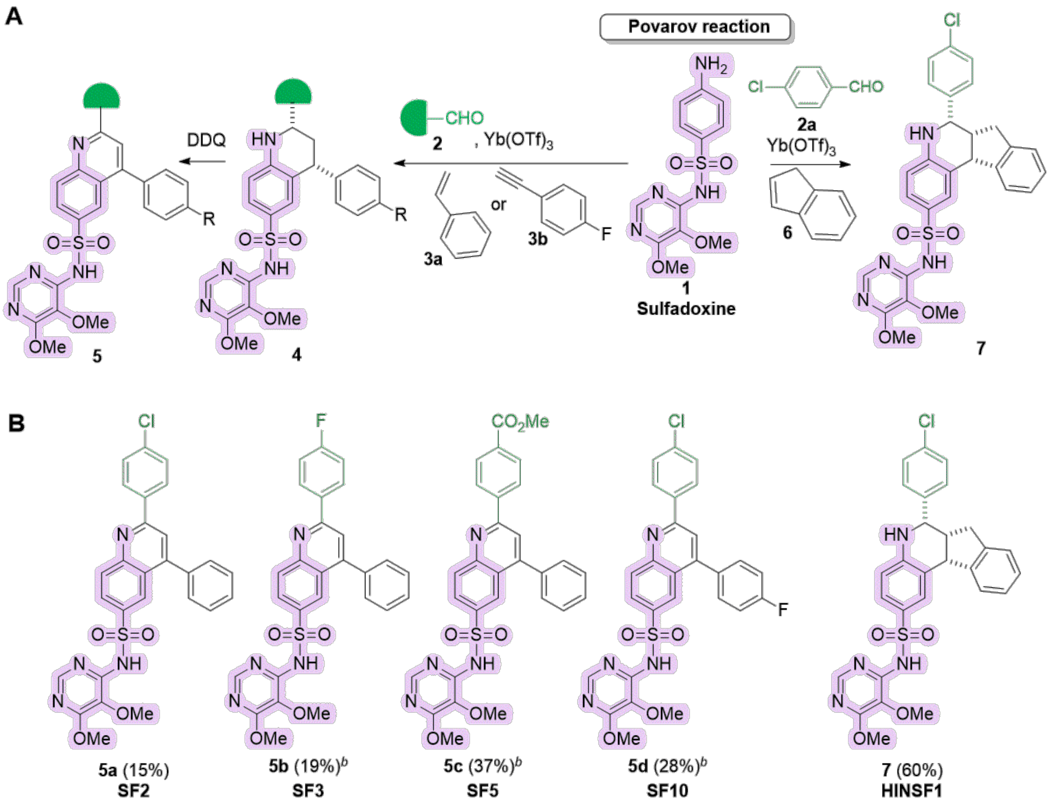

Figure S4. Synthesis of compounds targeting mtTOP1. **A**) Povarov reactions with aromatic amine **1**, aldehydes **2** and dienophiles **3** or **6**. **B**) Collection of heterocycles obtained by Povarov reactions. [b] Previously reported [1].

5. Supplementary S5

The structure of new compounds **SF2** and **HNSF1** was assigned on basis of NMR spectra and mass spectrometry (Figure S5).

A

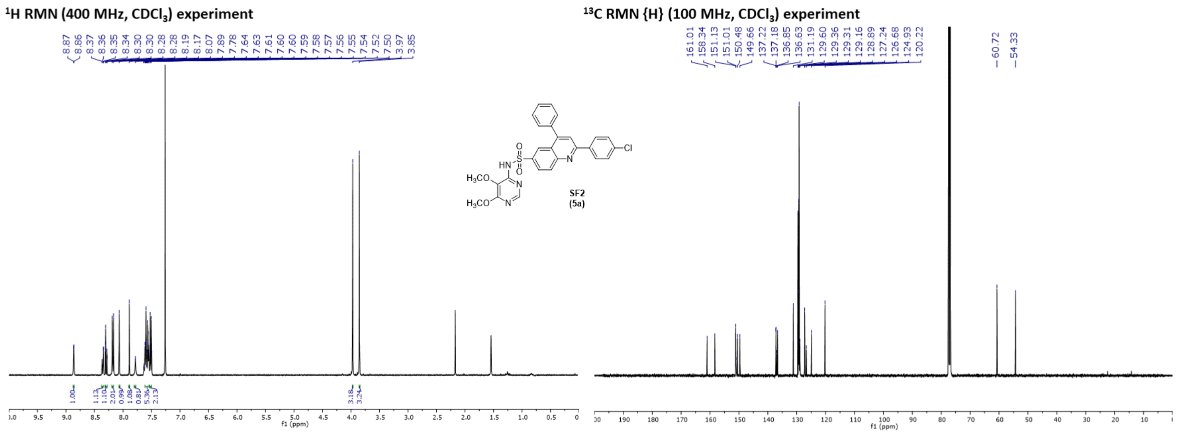

B

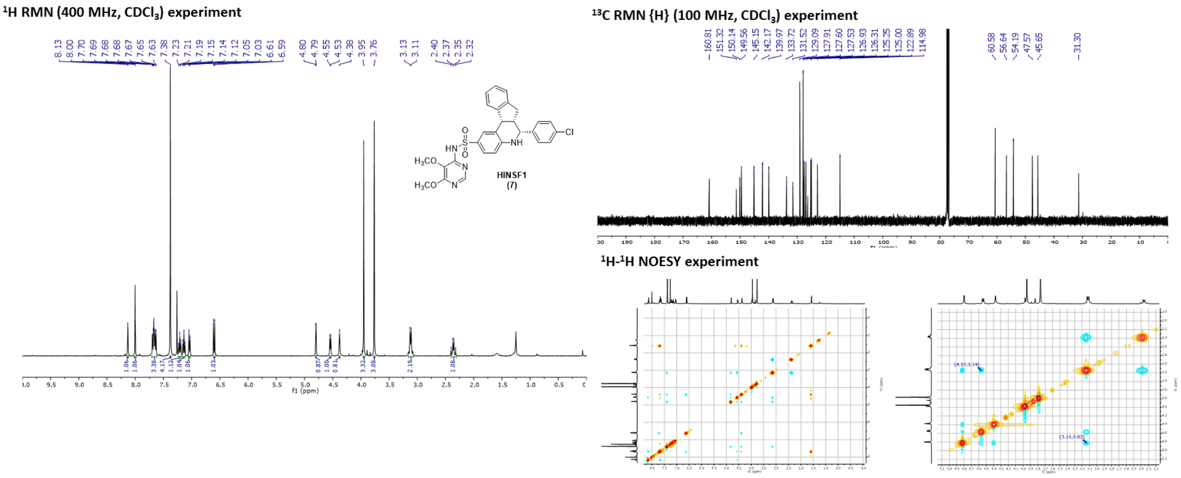

Figure S5. A) NMR spectra (^{1}H NMR and ^{13}C NMR) of compound **SF2**. B) NMR spectra (^{1}H NMR, ^{13}C NMR and ^{1}H-^{1}H NOESY experiments) of compound **HNSF1**.

Supplementary Materials and Methods

6.1. *Protein purification*

6.1.1. *hTOP1, LdTOP1, mpxvTOP1*

For expression and purification of hTOP1, LdTOP1 and mpxvTOP1, the RS190 yeast *Saccharomices cerevisiae* strain was transformed with the expression plasmid carrying the respective genes under the GAL1 promoter. The proteins were expressed, and purified enzyme were prepared as described previously [3,4]. The eluted proteins were analyzed on a 12% SDS PAGE and subsequently visualized with Coomassie Brilliant Blue staining.

6.1.2. *MsTOP1*

For expression and purification of MsTOP1, *E.coli* competent BL21 cells were transformed with the plasmid, pPVN123, expressing the MsTOP1 gene. The cells

were grown in 2xTY media supplemented with 100 μg/ml ampicillin and expression was induced in log phase at OD_{600} = 0.6 using 1 mM isopropyl b-D-1-thiogalactopyranoside (IPTG) at 37°C for 3 hours. Subsequently, the cells were harvested, resuspended in sonication buffer (20 mM Tris-HCl pH 7.5, 0.5 M NaCl, 10 mM EDTA, 10% glycerol, 1x complete protease inhibitor cocktail, 1/1000 PMSF, 10 mg/ml Lysozyme) and incubated on ice for 1 hour. The cells were then lysed by freeze and thawing in liquid N_2 three times followed by sonication 6x20 seconds with 100 seconds break (Branson Sonif 250). Cell debris were removed from the lysate by centrifugation at 10,000 rpm, 4°C for 30 minutes. The supernatant was transferred to new tubes and 5x volume of saturated ammoniumsulphate pH 7.5 was added and the lysate were let to precipitate overnight at 4°C. Following centrifugation at 10,000 rpm, 4°C, the precipitate was resuspended in 20 mM Tris-HCl pH 7.5, 500 mM NaCl, 1 mM DTT, and 10% glycerol, filtered through a 0.45 μm filter and loaded on a pre-equilibrated heparin column. The protein was eluted with 10 mM Tris-HCl pH 7.5, 1 M NaCl, 1 mM DTT, and 10% glycerol.

6.1.3. Phi29 polymerase

The synthetic gene for Phi29 polymerase (GenScript) was cloned into the pGEX vector generating in a recombinant N-terminal GST-tagged Phi29 polymerase expression plasmid.

For expression and purification of Phi29 polymerase, *E.coli* competent BL21 cells (Promega) were transformed with the plasmid. The cells were grown in 2xTY media supplemented with 100 μg/ml ampicillin and expression was induced in log phase at OD_{600} = 0.8 using 1 mM IPTG at 37°C for 2 hours. Subsequently, the cells were harvested, resuspended in sonication buffer (50 mM Tris-HCl pH 7.5, 2.5 M NaCl, 1 mM EDTA, 1 mM DTT, 10 mg/ml Lysozyme) and incubated on ice for 1 hour. The cells were then lysed by freeze and thawing in liquid N_2 followed by sonication. Afterwards, the lysate was mixed with 4% Steptomycin Sulfate and incubated for 1 hour at 4°C. Insoluble particles were removed by centriugation and the lysate was fitered using a 0.45 μm filter and subsequently loaded onto a pre-equilibrated GST Gravitrap column (GE Healthcare) following manufacture's instrutions. The column was washed in 10x columes of sonication buffer and protein eluted in 10x column volumes of elution buffer (10 mM Tris-HCl pH 8.5, 5 mM Glutathione, 500 mM NaCl) and collected in fractions. The eluted protein was analyzed on a 12% SDS PAGE and visualized by Coomassie Brilliant Blue staining. Finally, the protein fractions were dialysed against 50% glycerol, 0.5% Tween20, 1 mM DTT, and 0.5% NP40 at 4° overnight.

6.2. REEAD

REEAD was performed as in described in the main text, except that 40 μM and 80 μM of each compoud were used in the hTOP1 and LdTOP1 circularization reactions.

6.3. Synthesis of compounds

All reagents from commercial suppliers were used without further purification. All solvents were freshly distilled before use from appropriate drying agents. All other reagents were recrystallized or distilled when necessary. Reactions were performed under a dry nitrogen atmosphere. Analytical TLCs were performed with silica gel 60 F254 plates. Visualization was accomplished by UV light. Column chromatography was carried out using silica gel 60 (230-400 mesh ASTM) or neutral alumina (70-290 mesh ASTM). NMR spectra were obtained on a Bruker Avance 400 MHz spectrometer and recorded at 25 °C. Chemical shifts for ^{1}H NMR spectra are reported in ppm downfield from TMS, chemical shifts for ^{13}C NMR spectra are

recorded in ppm relative to internal chloroform (δ = 77.2 ppm for ^{13}C). Coupling constants (*J*) are reported in Hertz. The terms m, s, d, t, q refer to multiplet, singlet, doublet, triplet, quartet. ^{13}C NMR was broadband decoupled from hydrogen nuclei. High resolution mass spectra (HRMS) was measured by EI method with an Agilent LC-Q-TOF-MS 6520 spectrometer. Compounds **SF3**, **SF5** and **SF10** were prepared as previously described [1].

6.3.1. *Synthesis of N-(5,6-dimethoxypyrimidin-4-yl)-2-(4-chlorophenyl)-4-phenylquinoline-6 sulfonamide (***SF2***).*

To a suspension of sulfadoxine **1** (0.30 g, 1.0 mmol, 1.0 eq.), 4-chlorobenzaldehyde (0.136 g, 1.0 mmol, 1.0 eq.) and ytterbium triflate (0.120 g, 0.193 mmol, %20 mmol) in 5 mL of dry acetonitrile, and in presence of anhydrous $MgSO_4$ (800 mg) and styrene **3a** as dienophile (0.14 ml, 1.2 mmol) were stirred and heated to reflux for 48 h. The resulting mixture was stirred under nitrogen atmosphere at room temperature until TLC analysis indicated the disappearance of the starting materials. The solution was then diluted with dichloromethane (20 mL), washed with water (2 x 10 mL) and the aqueous layer was again extracted with dichloromethane (2 x 10 mL). The organic phase was dried over anhydrous $MgSO_4$, filtered and concentrated under vacuum. The corresponding crude residue was dissolved in $CHCl_3$ (15 mL), and DDQ (2 mmol) was added to the solution. The reaction mixture was stirred overnight at room temperature in an open vessel. A saturated aqueous solution of $NaHCO_3$ (10 mL) was added, the resulting mixture was extracted with dichloromethane (2 x 25 mL), dried over anhydrous $MgSO_4$, filtered and concentrated under reduced pressure. The crude residue obtained was purified by flash column chromatography on silica gel (hexane-ethyl acetate 80:20) to afford **SF2** in a 47% yield.

^{1}H-NMR (400 MHz, $CDCl_3$) δ 3.85 (s, 3H, OCH_3), 3.97 (s, 3H, OCH_3), 7.51 (d, $^3J_{HH}$ = 8.5 Hz, 2H, CH_{Ar}), 7.54 – 7.64 (m, 5H, CH_{Ar}), 7.78 (s, 1H, CH_{Ar}), 7.89 (s, 1H, NH), 8.07 (s, 1H, CH_{Ar}), 8.18 (d, $^3J_{HH}$ = 8.5 Hz, 2H, CH_{Ar}), 8.29 (dd, $^3J_{HH}$ = 8.9 Hz, 1H, CH_{Ar}), 8.36 (dd, $^3J_{HH}$ = 9.0 Hz, $^4J_{HH}$ = 2.1 Hz, 1H, CH_{Ar}), 8.86 (d, $^4J_{HH}$ = 2.1 Hz, 1H, CH_{Ar}) ppm.

^{13}C-NMR (100 MHz, $CDCl_3$) δ 54.3 (OCH_3), 60.7 (OCH_3), 120.2, 124.9, 126.7, 127.2, 128.9, 129.2, 129.3, 129.4, 129.6, 131.2, 136.6, 136.8, 137.2, 137.2, 149.6, 150.5, 151.0, 151.1, 158.3, 161.0 ppm.

HRMS: Calculated for $C_{27}H_{21}ClN_4O_4S$ $[M]^+$ 532.0972, found 532.0973.

6.3.2. *Synthesis of 6-(4-chlorophenyl)-N-(5,6-dimethoxypyrimidin-4-yl)-6,6a,7,11b-tetrahydro-5H-indeno[2,1-c]quinoline-2-sulfonamide (***HINSF1***).*

To a suspension of sulfadoxine **1** (0.30 g, 1.0 mmol, 1.0 eq.), 4-chlorobenzaldehyde (0.136 g, 1.0 mmol, 1.0 eq.) and ytterbium triflate (0.120 g, 0.16 mmol, %20 mmol) in 5 mL of dry acetonitrile, and in presence of anhydrous $MgSO_4$ (800 mg) and indene **6** as dienophile (0.169 ml, 1.2 mmol) were stirred and heated to reflux for 48 h. The resulting mixture was stirred under nitrogen atmosphere at room temperature until TLC analysis indicated the disappearance of the starting materials. The solution was then diluted with dichloromethane (20 mL), washed with water (2 x 10 mL) and the aqueous layer was again extracted with dichloromethane (2 x 10 mL). The organic phase was dried over anhydrous $MgSO_4$, filtered and concentrated under vacuum. The crude residue obtained was purified by flash column chromatography on silica gel (hexane-ethyl acetate 80:20) to afford **HINSF1** in a 60% yield.

^{1}H-NMR (400 MHz, $CDCl_3$) δ 2.36 (m, 1H, CH_2), 3.12 (m, 2H, CH + CH_2), 3.76 (s, 3H, OCH_3), 3.95 (s, 3H, OCH_3), 4.38 (s, 1H, NH), 4.54 (d, $^3J_{HH}$ = 6.6 Hz, 1H, CH-Ar), 4.80 (d, $^3J_{HH}$ = 2.6 Hz, 1H, CH-NH), 6.60 (d, $^3J_{HH}$ = 8.6 Hz, 1H, CH_{Ar}), 7.04 (d, $^3J_{HH}$ = 7.4 Hz,

1H, CH_{Ar}), 7.14 (t, $^3J_{HH}$ = 7.4 Hz, 1H, CH_{Ar}), 7.21 (t, $^3J_{HH}$ = 7.4 Hz, 1H, CH_{Ar}), 7.38 (s, 4H, CH_{Ar}), 7.61 – 7.71 (m, 3H, NH+ 2 CH_{Ar}), 8.00 (s, 1H, CH_{Ar}), 8.13 (s, 1H, CH_{Ar}) ppm.

^{13}C-NMR (100 MHz, $CDCl_3$) δ 31.3 (CH_2), 45.6, 47.6, 54.2 (OCH_3), 56.6, 60.5 (OCH_3), 115.0, 122.9, 125.0, 125.2, 126.3, 126.9, 127.5, 127.6, 127.9, 129.1, 131.5, 133.7, 140.0, 142.1, 145.1, 149.5, 150.1, 151.3, 160.8 ppm.

HRMS: Calculated for $C_{28}H_{25}ClN_4O_4S$ $[M]^+$ 548.1285, found 548.1285.

References

1. Trejo, A.; Masdeu, C.; Serrano-Pérez, I.; Pendrola, M.; Juanola, N.; Ghashghaei, O.; Jiménez-Galisteo, G.; Lavilla, R.; Palacios, F.; Alonso, C.; et al. Efficient AntiMycolata Agents by Increasing the Lipophilicity of Known Antibiotics through Multicomponent Reactions. *Antibiotics* **2023**, *12, 83*, doi: 10.3390/antibiotics12010083.
2. Selas, A.; Fuertes, M.; Melcón-Fernández, E.; Pérez-Pertejo, Y.; Reguera, R.M.; Balaña-Fouce, R.; Knudsen, B.R.; Palacios, F.; Alonso, C. Hybrid Quinolinyl Phosphonates as Heterocyclic Carboxylate Isosteres: Synthesis and Biological Evaluation against Topoisomerase 1b (Top1b). *Pharmaceuticals* **2021**, *14*, doi:10.3390/ph14080784.
3. Lisby, M.; Krogh, B.O.; Boege, F.; Westergaard, O.; Knudsen, B.R. Camptothecins Inhibit the Utilization of Hydrogen Peroxide in the Ligation Step of Topoisomerase I Catalysis. *Biochemistry* **1998**, *37*, 10815–10827, doi:10.1021/bi980757r.
4. Knudsen, B.R.; Straub, T.; Boege, F. Separation and Functional Analysis of Eukaryotic DNA Topoisomerases by Chromatography and Electrophoresis. *J Chromatogr B Biomed Appl* **1996**, *684*, 307-321.
